# Analyzing evaluation methods for large language models in the medical field: a scoping review

**DOI:** 10.1186/s12911-024-02709-7

**Published:** 2024-11-29

**Authors:** Junbok Lee, Sungkyung Park, Jaeyong Shin, Belong Cho

**Affiliations:** 1 Institute for Innovation in Digital Healthcare, Yonsei University, Seoul, Republic of Korea; 2 Department of Human Systems Medicine, Seoul National University College of Medicine, Seoul, Republic of Korea; 3 Department of Bigdata AI Management Information, Seoul National University of Science and Technology, Seoul, Republic of Korea; 4 Department of Preventive Medicine and Public Health, Yonsei University College of Medicine, 50-1, Yonsei-ro, Seodaemun-gu, Seoul, 03722 Republic of Korea; 5 Institute of Health Services Research, Yonsei University College of Medicine, Seoul, Korea; 6 Department of Family Medicine, Seoul National University Hospital, Seoul, Republic of Korea; 7 Department of Family Medicine, Seoul National University College of Medicine, 101 Daehak-ro, Jongno-gu, Seoul, 03080 Republic of Korea

**Keywords:** Large language model, LLM, Evaluation methods

## Abstract

**Background:**

Owing to the rapid growth in the popularity of Large Language Models (LLMs), various performance evaluation studies have been conducted to confirm their applicability in the medical field. However, there is still no clear framework for evaluating LLMs.

**Objective:**

This study reviews studies on LLM evaluations in the medical field and analyzes the research methods used in these studies. It aims to provide a reference for future researchers designing LLM studies.

**Methods & materials:**

We conducted a scoping review of three databases (PubMed, Embase, and MEDLINE) to identify LLM-related articles published between January 1, 2023, and September 30, 2023. We analyzed the types of methods, number of questions (queries), evaluators, repeat measurements, additional analysis methods, use of prompt engineering, and metrics other than accuracy.

**Results:**

A total of 142 articles met the inclusion criteria. LLM evaluation was primarily categorized as either providing test examinations (*n* = 53, 37.3%) or being evaluated by a medical professional (*n* = 80, 56.3%), with some hybrid cases (*n* = 5, 3.5%) or a combination of the two (*n* = 4, 2.8%). Most studies had 100 or fewer questions (*n* = 18, 29.0%), 15 (24.2%) performed repeated measurements, 18 (29.0%) performed additional analyses, and 8 (12.9%) used prompt engineering. For medical assessment, most studies used 50 or fewer queries (*n* = 54, 64.3%), had two evaluators (*n* = 43, 48.3%), and 14 (14.7%) used prompt engineering.

**Conclusions:**

More research is required regarding the application of LLMs in healthcare. Although previous studies have evaluated performance, future studies will likely focus on improving performance. A well-structured methodology is required for these studies to be conducted systematically.

**Supplementary Information:**

The online version contains supplementary material available at 10.1186/s12911-024-02709-7.

## Introduction

A Large Language Model (LLM) is a type of artificial intelligence (AI) designed to mimic human language processing using deep learning techniques trained on large amounts of textual data from various sources [1]. The rapid increase in the popularity of LLMs has led to numerous attempts to utilize them across different fields, with many demonstrating a significant level of competence [2]. LLMs are designed to respond to a wide range of topics, making them helpful tools for customer service, chatbots, and many other applications, hence the keen interest in their use in the medical field [3–6].

Several LLMs are currently accessible to researchers, each with unique features. OpenAI’s ChatGPT is widely used for its strong language understanding and generation capabilities [7]. Google’s Bard leverages vast search data to provide factual and accurate information [8]. Microsoft’s Bing Chat integrates chat with search for real-time information access [9]. In contrast, open-source LLMs like Meta’s LLaMA and Stanford’s Alpaca allow for customization and experimentation. While commercial models offer ease of use and technical support, open-source models provide flexibility and cost-effectiveness.

Various studies have been conducted in the medical field to verify the performance of LLMs. The following topics are being studied for the application of LLMs: (1) diagnostic and clinical decision support, (2) automation of medical records, (3) patient education and support, and (4) medical research and data analytics. LLMs can be utilized in diagnostic and clinical decision support to suggest possible diagnoses or treatment options based on a patient’s symptoms, medical history, and test results [10]. For medical record automation, LLMs have been studied for their potential to automatically organize and document patient encounters or generate explanatory materials to provide patients with information from their medical records [11]. Additionally, LLMs can enhance patient health literacy by explaining diseases, treatment options, and medication instructions in easy-to-understand language [12].

Only when LLMs perform at a human-like level in medical knowledge and reasoning assessments can users have sufficient confidence in their responses, and LLMs are useful in medical settings [13–15]. A framework has been proposed for LLM evaluation [16]. However, no clear methodology exists for evaluating LLMs in the medical field. In this study, we review the evaluation of LLMs in medicine. Based on these findings, we discuss essential points to consider when evaluating LLMs in medical applications.

## Methods & materials

### Study design

A scoping review aims to systematically synthesize knowledge within a defined area and explore and map key concepts, available evidence, and shortcomings of the existing research; it was determined to be the most appropriate method for this study [17, 18]. We considered several methodological approaches, including a systematic and narrative review. Systematic reviews focus narrowly on specific questions and similar methodologies, making them unsuitable for emerging technical areas with diverse evidence [19]. Narrative reviews offer the flexibility to synthesize diverse literature but lack the systematic rigor required to comprehensively map the research landscape and identify gaps in the literature [20]. The study followed the Preferred Reporting Items for Systematic Reviews and Meta-Analyses extension for Scoping Reviews (PRISMA-ScR) [21].

### Search strategy

We conducted a preliminary review to establish the search strategies. Initially, we searched the PubMed database using the keyword “Large Language Model*.” PubMed was utilized in the preliminary review of this study because it is a commonly suggested database to use when conducting a systematic review. A total of 498 articles were retrieved, and the review found that 73 articles that evaluated LLMs were identified.

**Fig. 1:**
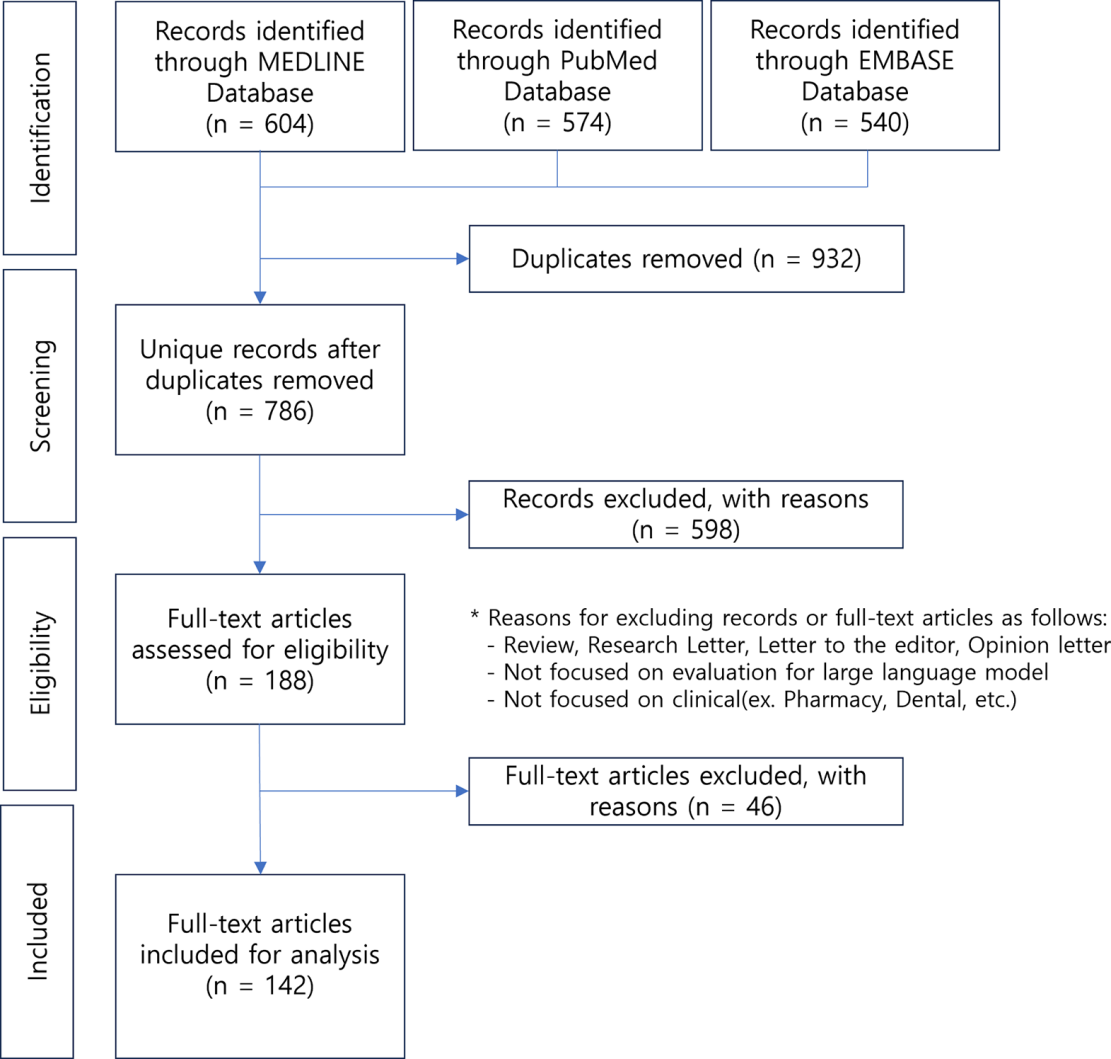
Analysis of evaluation by medical professionals

We found that papers assessing LLMs tended to use keywords such as “evaluation, assessment, performance, and comparison.” In addition, we included commercially available programs such as ChatGPT, Google’s Bard, and Microsoft’s Bing Chat in our search strategy. Given that the terminology for LLM gained prominence after 2023, we focused our search on the literature published between January 1, 2023, and September 30, 2023. We opted not to utilize MeSH terms, as the term LLM has only been in full use since early 2023; therefore, using MeSH terms may not reflect the latest research trends. After establishing the search strategy, we systematically searched MEDLINE, PubMed, and EMBASE (Fig. 1). The final search results were sent to EndNote to remove any duplicates. The search strategy used in this study is presented in Appendix Table 2.

### Selecting and screening studies

The screening process comprised two stages. Initially, articles were screened for relevance based on the information presented in the title and abstract, followed by a thorough assessment of inclusion based on the full text. Two authors (JB and SK) independently reviewed the articles. In cases of disagreement between the reviewers, a third independent reviewer (JY) was consulted to reach a consensus. For studies to be eligible for inclusion, they had to meet specific criteria, including being written in English and addressing the evaluation of LLMs in healthcare settings. We excluded publications such as conference abstracts, editorials, reviews, research letters, letters to the editor, and opinion letters. Articles in the pharmacy and dentistry fields were excluded from the screening process. A comprehensive list of inclusion and exclusion criteria can be found in the appendix.

### Extracting and analyzing the data

We summarized information regarding the evaluation method, type of LLMs, and medical specialty for the studies included in the review.

For test-based evaluation, we analyzed the number of questions and repeated measurements, use of prompt engineering (e.g., few-shot learning, role-based prompting), additional analysis, whether the questions were analyzed for difficulty, and the primary outcomes. The number of repeated measurements refers to administering the same prompt more than once to evaluate the consistency of the responses generated by the LLMs. In prompt engineering, few-shot learning refers to a methodology where a small number of examples are provided within a prompt to guide the model in learning patterns and generating correct answers, while role-based prompting means designing prompts so that the model adopts a specific role when answering. Additional analysis refers to whether further assessments were conducted beyond accuracy, including the overall adequacy of logical reasoning and evidence provided, the frequency of hallucinations, and the error types made in incorrect responses. For the LLM evaluation by medical professionals, we analyzed the number of queries, number of repeated measurements, number of evaluators, use of prompt engineering, evaluation tools and sources, evaluation items, and scales.

In contrast to studies that conducted both test-based evaluations and assessments by healthcare professionals, some studies removed the selection of test questions and had medical professionals evaluate the LLM responses directly, a method referred to as a hybrid approach. Research that used both methods or a hybrid approach was analyzed by including them in test-based evaluations and evaluations by medical professionals.

### Statistical analysis

We conducted a Kruskal-Wallis test to compare the performance differences among the four LLMs (GPT-3.5, GPT-4, Bing Chat, and Bard). This nonparametric method was chosen due to the small sample size for each model evaluation. Following this, post hoc pairwise comparisons were conducted using the Mann-Whitney U test with Bonferroni correction applied to account for multiple comparisons, identifying which specific pairs of models show significant differences. For all statistical tests, a significance level of 0.05 was used. The analysis was performed using STATA 16 (StataCorp LLC, College Station, TX, USA).

## Results

### Overview

Following the Preferred Reporting Items for Systematic Reviews and Meta-Analyses (PRISMA) flowchart (Fig. 1), four review steps were performed: identification, screening, eligibility assessment, and final consensus. The initial search retrieved 1,718 unique articles. Automatic deduplication using ENDNOTE removed 894 articles, 38 of which were identified as manual duplicates. After reviewing all the abstracts, 598 (34.8%) were excluded based on the exclusion criteria. A total of 188 (10.9%) articles underwent a full-text review, of which 46 (2.7%) did not meet the inclusion criteria, leaving 142 (8.3%) for final inclusion and analysis. The Appendix Tables 8 and 9 provide data excerpts from the papers.

### Characteristics of published literature

The effectiveness of the LLMs was assessed in two ways: evaluation based on test examination (*n* = 53, 37.3%) [22–74] and evaluation by medical professionals (*n* = 80, 56.3%) [75–154]. Others used a combination of both (*n* = 4, 2.8%) [155–158] or a hybrid approach to evaluate the LLMs’ responses to the test examination (*n* = 5, 3.5%) [159–163] (Table 1).

**Table 1:** LLMs evaluation methods

Methods	(*n* = 142)
	*N* (%)
Test questions	53 (37.3)
Expert evaluation	80 (56.3)
Hybrid approach	5 (3.5)
Both	4 (2.8)

Articles evaluating LLM often used several models instead of only one (*n* = 88, 54%). A total of 218 LLMs were used in 142 studies, including this study (Table 2). The most common LLM used was the Open AI’s GPT-3.5 (*n* = 114, 52.3%), followed by GPT-4 (*n* = 65, 29.8%). Google’s Bard (*n* = 15, 6.9%) and Microsoft’s Bing Chat (*n* = 12, 5.5%) were the third and fourth most common. A few models were developed by fine-tuning the models (*n* = 3, 1.4%).

**Table 2:** LLMs used in the evaluation

Language Model	Expert evaluation	Test questions	Hybrid approach	Both	Total
GPT-3.5	61	45	4	4	114 (52.1)
GPT-4	30	34	1	1	66 (30.1)
Bard	8	6	-	1	15 (6.8)
Bing Chat	7	4	-	1	12 (5.5)
ETC (GPT-3, GPT-2)	2	7	-	-	9 (4.1)
Fine tuning	3	-	-	-	3 (1.4)
**Total**	**111**	**96**	**5**	**7**	**219 (100.0)**

In terms of medical specialties, internal medicine (*n* = 23, 16.2%) was the most common medical specialty to which the LLMs were applied (Table 3), followed by radiology (*n* = 16, 11.3%) and ophthalmology (*n* = 15, 10.6%). Regarding Internal Medicine, Cardiovascular Disease and Gastroenterology had the highest number of LLM evaluation papers (six each). In addition, some general practices did not belong to a specific medical department (*n* = 19, 13.4%) and were mainly validated by examination. We derived suggestions for systematically designing studies evaluating LLMs in healthcare based on our findings.

**Table 3:** LLMs used in medical specialties

Medical Specialty	(*n* = 142)	
	*N*	(%)
Anesthesiology	1	(0.7)
Dermatology	4	(2.8)
Emergency Medicine	3	(2.1)
Family Medicine	1	(0.7)
Internal Medicine	23	(16.2)
Neurological Surgery	5	(3.5)
Obstetrics and Gynecology	6	(4.2)
Ophthalmology	15	(10.6)
Orthopaedic Surgery	9	(6.3)
Otolaryngology – Head and Neck Surgery	7	(4.9)
Pathology	4	(2.8)
Pediatrics	1	(0.7)
Plastic Surgery	4	(2.8)
Psychiatry and Neurology	6	(4.2)
Radiology	16	(11.3)
Surgery	4	(2.8)
Thoracic Surgery	3	(2.1)
Urology	8	(5.6)
General Practice	19	(13.4)
ETC (clinical informatics, nursing)	3	(2.1)

### Evaluation based on test examination

Regarding the number of questions used for evaluation, less than 100 were the most common (*n* = 18, 29.0%), followed by 200–300 (*n* = 14, 22.6%), then 100–200 (*n* = 11, 17.7%), and 500 or more (*n* = 11, 17.7%) (Table 4). Regarding repeated measures, about three-quarters of the studies did not perform any repeated measures (*n* = 47, 75.8%). Five papers (8.1%) did this twice, six papers did it three times (9.7%), and four papers did it four or more times (6.5%). Eight (12.9%) studies applied prompt engineering to improve the LLM performance. Seven studies employed role-based prompting, while one used the few-shot learning method with examples. Eighteen papers (29.0%) conducted additional analyses beyond simply measuring correct responses to the questions, and fourteen papers (14%) conducted analyses based on question difficulty.

**Table 4:** Analysis of evaluation based on test examinations

	(*n*=62)	
	**N**	**(%)**
**Number of questions**		
1–100	18	(29.0)
101–200	11	(17.7)
201–300	14	(22.6)
301–400	5	(8.1)
401–500	3	(4.8)
501-	11	(17.7)
**Number of repeated measurements**		
0	47	(75.8)
2	5	(8.1)
3	6	(9.7)
above 4	4	(6.5)
**Prompt engineering**		
Yes	8	(12.9)
No	54	(87.1)
**Additional analysis**		
Yes	18	(29.0)
No	44	(71.0)
**Difficulty**		
Yes	14	(22.6)
No	48	(77.4)

**Fig. 2:**
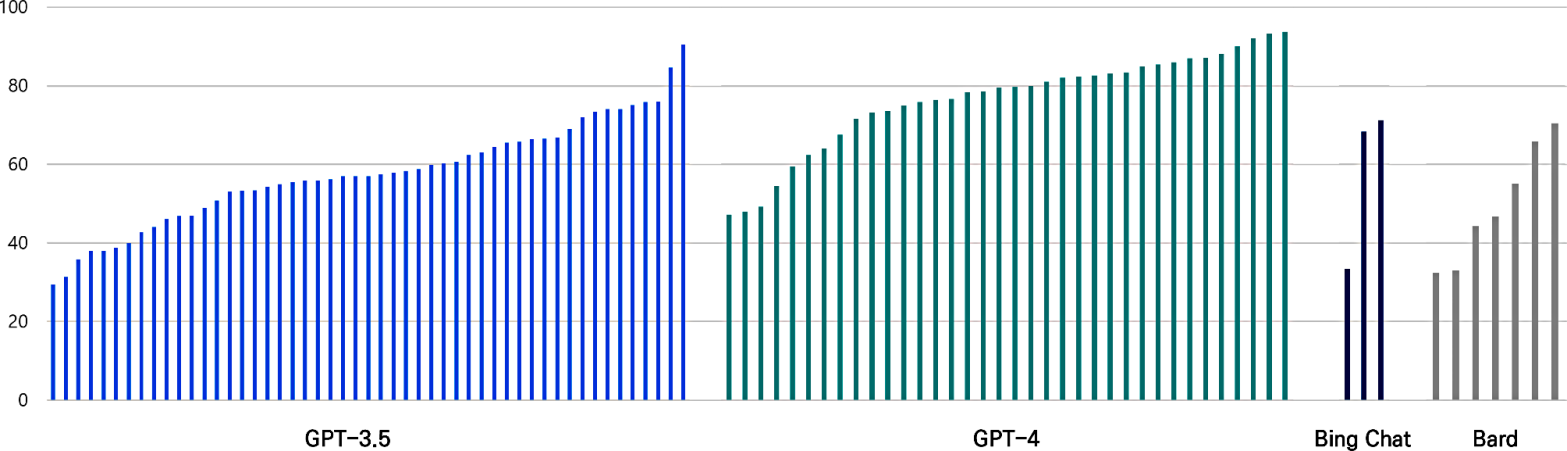
Performance of GPT-3.5, GPT-4, Bing Chat and Bard

The performance of LLMs is illustrated in Fig. 2. Among the models evaluated, GPT-4 exhibited the highest accuracy (mean: 76.47, median: 79.65, SD: 12.57, IQR: 12.30), while GPT-3.5 (mean: 57.62, median: 57.00, SD: 13.26, IQR: 16.25) and Bing Chat (mean: 57.61, median: 68.33, SD: 21.10, IQR: 18.95) demonstrated lower accuracy scores. Bard had the lowest accuracy (mean: 49.63, median: 46.67, SD: 14.94, IQR: 21.82. We conducted a Kruskal-Wallis test to assess the differences in performance across the models statistically. The analysis revealed significant model differences (H = 35.51, *p* < 0.001). Post-hoc analysis using the Mann-Whitney U test indicated significant differences between GPT-4 and GPT-3.5 (z = -5.50, *p* < 0.001), GPT-4 and Bard (z = -3.52, *p* < 0.001), and GPT-4 and Bing Chat (z = -2.00, *p* = 0.045). However, no significant differences were found between GPT-3.5 and Bard, GPT-3.5 and Bing Chat, or Bard and Bing Chat (*p* > 0.05). It is important to note that the number of studies involving Bard and Bing Chat is limited, and results should be interpreted cautiously.

### Evaluation by medical professionals

Regarding the number of queries, 50 or fewer was the most common (*n* = 54, 64.3%), followed by 50–100 (*n* = 14, 16.7%), then 151–200 (*n* = 7, 8.3%), 101–150 (*n* = 6, 7.1%), and 150–200 (*n* = 7, 8.3%) (Table 5). Regarding repeated measures, about 70% of the studies did not perform any repeated measures (*n* = 63, 70.8%). Eleven papers (12.4%) did it twice, ten papers did it three times (11.2%), and five papers did it five times (5.6%). Among the experts who evaluated the LLMs, two were the most common (*n* = 43, 48.3%), followed by 3 (*n* = 13, 14.6) and by 1 (*n* = 5). Thirteen (14.7%) studies applied prompt engineering to improve the LLM performance.

**Table 5:** Analysis of evaluation by medical professionals

	(*n* = 89)	
**Number of queries**	**N**	**(%)**
1–50	54	(64.3)
51–100	14	(16.7)
101–150	6	(7.1)
151–200	7	(8.3)
201 above	3	(3.6)
**Number of repeat measurements**	**N**	**(%)**
0	63	(70.8)
2	11	(12.4)
3	10	(11.2)
5	5	(5.6)
**Number of evaluators**	**N**	**(%)**
1	5	(5.6)
2	43	(48.3)
3	13	(14.6)
4	3	(3.4)
5 above	17	(19.1)
(not indicated)	8	(9.0)
**Prompt Tuning**	**N**	**(%)**
None	76	(85.4)
Role-based prompting	6	(6.7)
Few shots learning	2	(2.3)
Explain context	2	(2.3)
Template	3	(3.4)

In addition to accuracy, we identified several metrics used for LLM evaluation (Table 6). The papers we reviewed evaluated whether the LLM’s responses were in concord with guidelines or expert opinions (*n* = 12) or whether the responses were appropriate (*n* = 9), complete (*n* = 8), or of high quality (*n* = 8). A few studies also assessed the safety (*n* = 5) or readability (*n* = 3) of the responses, as well as their clarity (*n* = 3).

**Table 6:** Metrics used for LLMs evaluation (except accuracy)

Metric	No.
Concordance / Agreement with guidelines or experts	12
Appropriateness	9
Completeness	8
Quality	8
Reproducibility	7
Safety, Extent of harm	6
Readability	5
Relevance	5
Clarity	3
Acceptability	2
Comprehensiveness	2
Currency of information	2
Efficacy	2
Empathetic	2
Helpfulness	2
Reliability	2
Understandability	2
Usefulness	2
Preference	1
Satisfaction	1
Specificity	1
Validity of references	1
Over-conclusiveness	1
Supplemental information	1
Objectivity	1
Bias	1
Adaptiveness	1

## Discussion

**Fig. 3:**
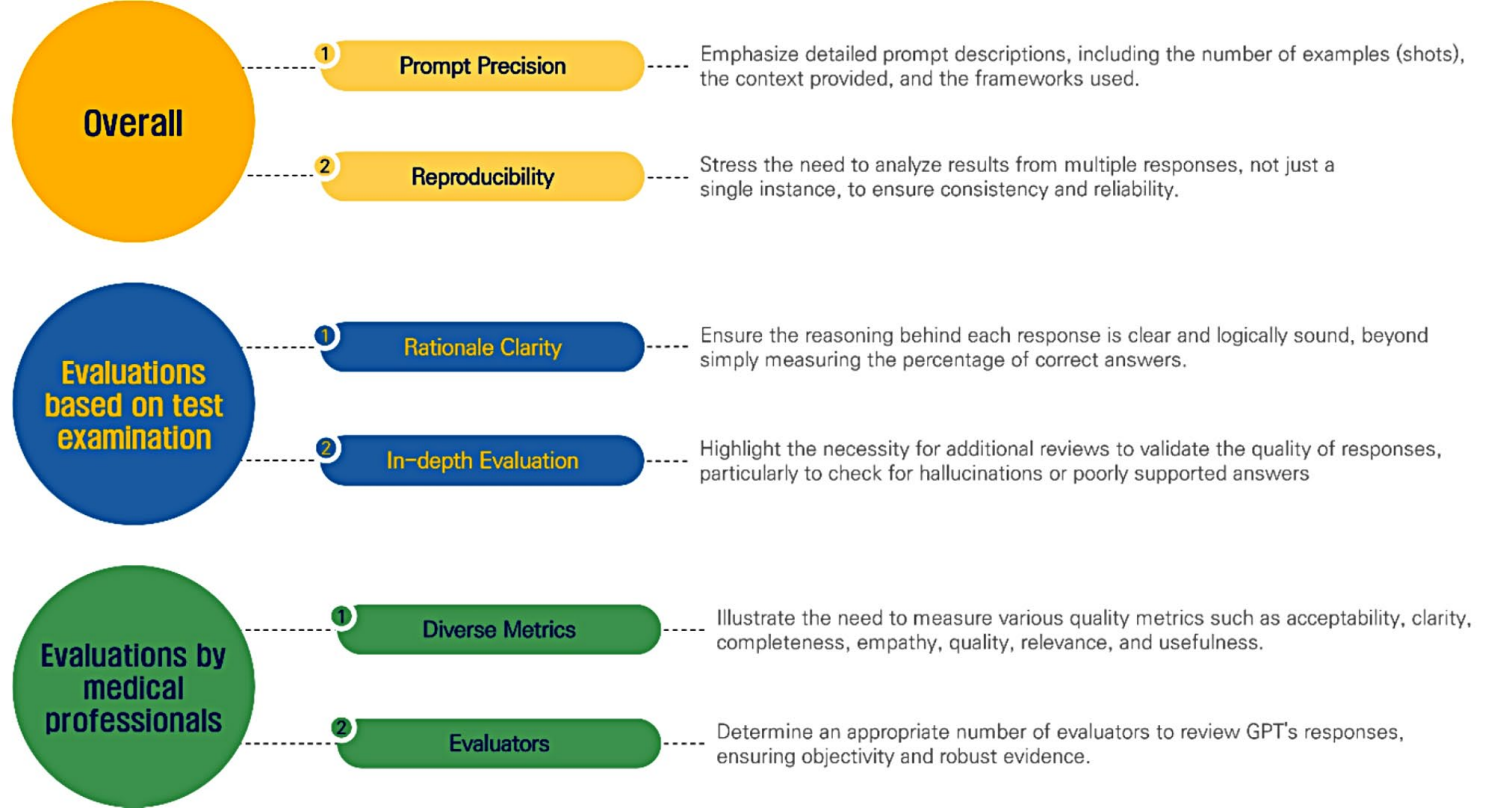
Improving the evaluation of LLMs

This study aimed to analyze methods for evaluating LLMs in medicine. For LLM performance evaluation, two main methods were used: evaluation based on test examinations and evaluation by medical professionals. In addition, there is a method that uses both methods together and a hybrid method. Evaluation based on a test examination was used to evaluate LLM performance according to the medical specialty, and evaluation by medical professionals was mainly used when LLMs were utilized for particular purposes, such as clinical decision support or answering questions. Based on our findings, we derived suggestions for systematically designing studies evaluating LLMs in healthcare (Fig. 3).

### Better evaluations based on test examination

For evaluations based on a test examination, beyond simply presenting the percentage of correct responses, studies should also be reviewed to ensure the evidence is presented. Some studies have performed additional evaluations, such as checking for concordance with the proposed correct answer or reviewing responses to ensure that they are well-founded with an appropriate rationale; however, the number of such studies remains relatively limited [25, 26, 34, 37, 39, 41, 46]. Given the high number of reports on hallucinations in the LLMs, additional reviews are needed to ensure that hallucinations, poorly supported answers, and reasoning are accurately reported [164–166].

In a test examination-based evaluation, the difficulty of the questions is a critical factor to consider. Approximately a quarter of the papers included in this study have evaluated performance based on question difficulty. By analyzing performance about difficulty, researchers can determine the level of complexity at which the LLMs perform optimally or begin to degrade. It is also essential to assess how the LLMs respond to varying difficulty levels, as clinical settings often present questions of differing complexities. Finally, such analysis can guide future research to improve model performance through prompt engineering or fine-tuning approaches. Developing an evaluation framework to verify the reasoning behind LLMs is required. Some studies analyzed incorrect answers by categorizing them as logical, informational, or statistical errors, and one study proposed a CVSA (Concordance, Validity, Safety, and Accuracy) model [49].

### Better evaluations by medical professionals

Most studies on LLMs have measured the accuracy. However, it is also necessary to measure various other metrics. In addition to accuracy, the reviewed studies measured concordance with guidelines or expert opinion and the responses’ appropriateness, completeness, quality, safety, readability, and clarity. Although it may vary from one medical field to another or depending on the purpose of the study, an evaluation frame or guideline for the evaluation of LLMs is also needed. Because different people may have different ideas about a term, researchers must precisely describe what they measure and present the scale.

In addition, most studies used two people to evaluate the LLM responses, but two people should not be considered an appropriate number for evaluating the LLM performance. When future guidelines for LLM evaluation are developed, an appropriate number of evaluators should be considered to ensure representativeness.

### Need for considering reproducibility

A study design that considers reproducibility is required. Some studies have performed two or three repeated measurements to ensure reproducibility. Because the LLMs do not always provide the same response, we believe it is better to draw results and analyze them for multiple responses rather than just one. Studies that have validated reproducibility have reported reproducibility rates of 90–100% [88, 89, 113, 142]. While 5–10% may not seem like a lot, given the specificity of the medical field, we believe that reproducibility should be considered.

### Need for accurate prompt descriptions

Lastly, an accurate description of the prompts is necessary. The LLMs can produce very different results depending on how the prompts are written. Various engineering methods have been proposed to improve the LLM performance. Therefore, researchers must be precise regarding prompting. For example, it is necessary to be precise about the number of examples for few-shot learning, whether roles-based prompting is given, and the use of frameworks. Some studies did not provide supplementary materials or figures for the prompts, so checking how the prompts were written was impossible. Therefore, it would be helpful for follow-up studies to provide supplementary materials about the writing of the prompts. Additionally, it would be helpful for researchers to maintain a version of the prompts when recording or revising a study.

## Limitations

The limitations of this study were as follows. First, some of the LLM evaluation studies may not have been included due to the scope of our search strategy. Specifically, we did not include open-source models such as LLAMA or ALPACA, which may have led to the omission of relevant studies. Additionally, while we utilized representative databases for our search, similar research may exist in other databases not included in our review. However, we believe the large number of reviewed papers (142) mitigates this limitation. In future research, including more databases and expanding the search strategy to cover additional models could help address these limitations. Additionally, the scoping review required a qualitative evaluation of the studies, but this was not performed because there is no established evaluation methodology for LLMs. We hope this study will contribute to developing criteria for the qualitative evaluation of LLM research.

## Conclusion

LLMs are being applied in various ways, and we expect them to become more advanced. They have several potential applications in medicine. However, according to the medical field’s characteristics, accuracy is critical, and incorrect information should not be provided to patients. It is necessary to increase reliability through various evaluations before LLMs can be used in the medical field. Future studies should conduct additional analyses to examine factors such as reasoning ability, hallucinations, and the difficulty of test questions. Moreover, these studies should consider applying metrics beyond accuracy and ensure reproducibility.

## Electronic supplementary material

Below is the link to the electronic supplementary material.

Supplementary Material 1

## Data Availability

The data from this study is available in the supplementary file. Additional data can be requested from the corresponding author if needed.
