# Supplementary material for: Analyzing evaluation methods for large language models in the medical field: a scoping review
Source: BMC Med Inform Decis Mak. 2024 Nov 29;24:366. doi: 10.1186/s12911-024-02709-7 (PMC11606129; doi:10.1186/s12911-024-02709-7)
Supplement: Supplementary file 1 — Supplementary Material 1 [file 12911_2024_2709_MOESM1_ESM.docx]

**Appendix Table 1. Preferred Reporting Items for Systematic reviews and Meta-Analyses extension for Scoping Reviews (PRISMA-ScR) Checklist**

| **SECTION** | **ITEM** | **PRISMA-ScR CHECKLIST ITEM** | **REPORTED ON PAGE #** |
| --- | --- | --- | --- |
| **TITLE** | | | |
| Title | 1 | Identify the report as a scoping review. | 1 |
| **ABSTRACT** | | | |
| Structured summary | 2 | Provide a structured summary that includes (as applicable): background, objectives, eligibility criteria, sources of evidence, charting methods, results, and conclusions that relate to the review questions and objectives. | 3 |
| **INTRODUCTION** | | | |
| Rationale | 3 | Describe the rationale for the review in the context of what is already known. Explain why the review questions/objectives lend themselves to a scoping review approach. | 4-5 |
| Objectives | 4 | Provide an explicit statement of the questions and objectives being addressed with reference to their key elements (e.g., population or participants, concepts, and context) or other relevant key elements used to conceptualize the review questions and/or objectives. | 4-5 |
| **METHODS** | | | |
| Protocol and registration | 5 | Indicate whether a review protocol exists; state if and where it can be accessed (e.g., a Web address); and if available, provide registration information, including the registration number. | N/A |
| Eligibility criteria | 6 | Specify characteristics of the sources of evidence used as eligibility criteria (e.g., years considered, language, and publication status), and provide a rationale. | 5, Appendix Table 2 |
| Information sources | 7 | Describe all information sources in the search (e.g., databases with dates of coverage and contact with authors to identify additional sources), as well as the date the most recent search was executed. | 5 |
| Search | 8 | Present the full electronic search strategy for at least 1 database, including any limits used, such that it could be repeated. | 5, Appendix Table 3 |
| Selection of sources of evidence | 9 | State the process for selecting sources of evidence (i.e., screening and eligibility) included in the scoping review. | 6 |
| Data charting process | 10 | Describe the methods of charting data from the included sources of evidence (e.g., calibrated forms or forms that have been tested by the team before their use, and whether data charting was done independently or in duplicate) and any processes for obtaining and confirming data from investigators. | 6 |
| Data items | 11 | List and define all variables for which data were sought and any assumptions and simplifications made. | 6-7 |
| Critical appraisal of individual sources of evidence | 12 | If done, provide a rationale for conducting a critical appraisal of included sources of evidence; describe the methods used and how this information was used in any data synthesis (if appropriate). | N/A |
| Synthesis of results | 13 | Describe the methods of handling and summarizing the data that were charted. | 6-7 |
| **RESULTS** | | | |
| Selection of sources of evidence | 14 | Give numbers of sources of evidence screened, assessed for eligibility, and included in the review, with reasons for exclusions at each stage, ideally using a flow diagram. | 8 |
| Characteristics of sources of evidence | 15 | For each source of evidence, present characteristics for which data were charted and provide the citations. | 8-9 |
| Critical appraisal within sources of evidence | 16 | If done, present data on critical appraisal of included sources of evidence (see item 12). | N/A |
| Results of individual sources of evidence | 17 | For each included source of evidence, present the relevant data that were charted that relate to the review questions and objectives. | 8-10 |
| Synthesis of results | 18 | Summarize and/or present the charting results as they relate to the review questions and objectives. | 8-10 |
| **DISCUSSION** | | | |
| Summary of evidence | 19 | Summarize the main results (including an overview of concepts, themes, and types of evidence available), link to the review questions and objectives, and consider the relevance to key groups. | 11 |
| Limitations | 20 | Discuss the limitations of the scoping review process. | 13 |
| Conclusions | 21 | Provide a general interpretation of the results with respect to the review questions and objectives, as well as potential implications and/or next steps. | 13 |
| **FUNDING** | | | |
| Funding | 22 | Describe sources of funding for the included sources of evidence, as well as sources of funding for the scoping review. Describe the role of the funders of the scoping review. | N/A |

**Appendix Table 2. Inclusion and exclusion criteria**

|  | Inclusion criteria | Exclusion criteria |
| --- | --- | --- |
| Search string | - Articles searched based on the search string in the following databases (PubMed, Medline, Embase) | - Duplicates |
| Retrieved article type | - Original peer-reviewed articles in scientific journals - Evaluation of large language models - Full text available | - Review articles - Research letter - Letter to the editor - Opinion letter |
| Fields | - Clinical | - Pharmacy - Dentistry |

**Appendix Table 3. Search strategy for study selection**

| #1 | "Large Language Model*" OR "LLM*" OR "ChatGPT" OR "Google Bard" OR "Bing Chat” |
| --- | --- |
| #2 | "evaluat*" OR "assess*" OR "compar*" OR “perform*” |
| #3 | #1 AND #2 |

**Appendix Table 4. References of the LLM models**

| **Language Model** | **N** | **Reference** |
| --- | --- | --- |
| **Evaluation based on test examination** | **96** |  |
| GPT-3.5 | 45 | Ali et al [22], Ali et al [23], Antaki et al [24], Bhayana et al [25], Cai et al [26], Cohen et al [28], Cuthbert et al [29], Friederichs et al [31], Gencer et al [32], Giannos et al [33], Gilson et al [34], Guigue et al [36], Gupta et al [37], Hoch et al [38], Holmes et al [39], Hopkins et al [40], Huang et al [41], Humar et al [42], Hurley et al [43], Kaneda et al [44], Kumah-Crystal et al [45], Kung et al [46], Lewandowski et al [47], Li et al [48], Lum et al [50], Madrid-García et al [51], Massey et al [52], Meo et al [53], Mihalache et al [54], Moshirfar et al [55], Noda et al [56], Oh et al [57], Oztermeli et al [58], Passby et al [59], Roos et al [61], Rosoł et al [62], Schubert et al [64], Shetty et al [65], Smith et al [66], Taira et al [68], Takagi et al [69], Tanaka et al [70], Teebagy et al [71], Thirunavukarasu et al [72], Wang et al [74] |
| GPT-4 | 34 | Alietal et al [22], Alietal et al [23], Antakietal et al [24], Caietal et al [26], Genceretal et al [32], Giannosetal et al [33], Guerraetal et al [35], Holmesetal et al [39], Huangetal et al [41], Hurleyetal et al [43], Kanedaetal et al [44], Kungetal et al [46], Lewandowskietal et al [47], Lietal et al [48], Longetal et al [49], Madrid-Garcíaetal et al [51], Masseyetal et al [52], Moshirfaretal et al [55], Nodaetal et al [56], Ohetal et al [57], Passbyetal et al [59], Patiletal et al [60], Roosetal et al [61], Rosołetal et al [62], Saadetal et al [63], Schubertetal et al [64], Shettyetal et al [65], Smithetal et al [66], Suchmanetal et al [67], Takagietal et al [69], Tanakaetal et al [70], Teebagyetal et al [71], Valdezetal et al [73], Wangetal et al [74] |
| Bard | 6 | Alietal et al [23], Holmesetal et al [39], Hurleyetal et al [43], Nodaetal et al [56], Patiletal et al [60], Smithetal et al [66] |
| Bing Chat | 4 | Caietal et al [26], Chenetal et al [27], Roosetal et al [61], Smithetal et al [66] |
| ETC | 7 | Deebeletal et al [30], Gilsonetal et al [34], Holmesetal et al [39], Lietal et al [48], Shettyetal et al [65], Suchmanetal et al [67], Valdezetal et al [73] |
| **Evaluation by medical professionals** | **111** |  |
| GPT-3.5 | 61 | Abi-Rafeh et al [75], Ali et al [76], Allahqoli et al [77], Athavale et al [78], Ayers et al [79], Ayoub et al [80], Bellinger et al [82], Bernstein et al [84], Biswas et al [86], Caglar et al [88], Cakir et al [89], Chen et al [90], Chiesa-Estomba et al [91], Clough et al [92], Cocci et al [93], Coskun et al [94], Coskun et al [95], Davis et al [96], Delsoz et al [97], Delsoz et al [98], Duey et al [99], Fink et al [100], Haemmerli et al [102], Henson et al [103], Hirosawa et al [104], Hristidis et al [106], Hung et al [108], Johnson et al [109], Kao et al [111], King et al [112], King et al [113], Kiyohara et al [114], Kumari et al [117], Kuroiwa et al [118], Kusunose et al [119], Lahat et al [120], Lim et al [121], Liu et al [122], Lukac et al [123], Lyu et al [125], Mika et al [126], Mishra et al [127], Nakaura et al [129], O'Hagan et al [130], Rahsepar et al [132], Rao et al [133], Rao et al [134], Rau et al [135], Rogasch et al [137], Rojas-Carabali et al [138], Russe et al [139], Zúñiga Salazar et al [140], Samaan et al [141], Samaan et al [142], Sarbay et al [143], Shao et al [144], Stevenson et al [145], Sütcüoğlu et al [146], Uz et al [149], Whiles et al [153], Yeo et al [154] |
| GPT-4 | 30 | Athavale et al [78], Barash et al [81], Benirschke et al [83], Cadamuro et al [87], Coskun et al [95], Delsoz et al [97], Duey et al [99], Fink et al [100], Gorelik et al [101], Hu et al [107], Kaarre et al [110], King et al [112], King et al [113], Kiyohara et al [114], Krusche et al [115], Lim et al [121], Lyons et al [124], Lyu et al [125], Momenaei et al [128], O'Hagan et al [130], Qu et al [131], Rao et al [133], Rau et al [135], Reese et al [136], Rojas-Carabali et al [138], Russe et al [139], Suthar et al [147], Ueda et al [148], Vaira et al [150], Wang et al [152] |
| Bard | 8 | Coskun et al [95], Hirosawa et al [105], Kiyohara et al [114], Kumari et al [117], Lim et al [121], Rahsepar et al [132], Zúñiga Salazar et al [140], Stevenson et al [145] |
| Bing Chat | 7 | Birkun et al [85], Coskun et al [95], Kuckelman et al [116], Kumari et al [117], Lyons et al [124], Nakaura et al [129], Zúñiga Salazar et al [140] |
| ETC | 2 | Nakaura et al [129], Wagner et al [151] |
| Fine tuning | 3 | Athavale et al [78], Rau et al [135], Russe et al [139] |
| **Hybrid approach** | **5** |  |
| GPT-3.5 | 4 | Beaulieu-Jones et al [159], Huynh et al [161], Kung et al [162], Strong et al [163] |
| GPT-4 | 1 | Fang et al [160] |
| **Both** | **7** |  |
| GPT-3.5 | 4 | Angel et al [155], Chervenak et al [156], Copeland-Halperin et al [157], Harskamp et al [158] |
| GPT-4 | 1 | Angel et al [155] |
| Bard | 1 | Angel et al [155] |
| Bing Chat | 1 | Copeland-Halperin et al [157] |

**Appendix Table 5. References of medical specialties**

| **Medical Specialty** |  |  | **N** | **Reference** |
| --- | --- | --- | --- | --- |
| Anesthesiology |  |  | 1 | Angel et al [155] |
| Dermatology |  |  | 4 | Lewandowskietal et al [47], Passbyetal et al [59], Shettyetal et al [65], O'Hagan et al [130] |
| Emergency Medicine |  |  | 3 | Smithetal et al [66], Zúñiga Salazar et al [140], Sarbay et al [143] |
| Family Medicine |  |  | 1 | Huangetal et al [41] |
| Internal Medicine |  |  | 23 | Nodaetal et al [56], Suchmanetal et al [67], Valdezetal et al [73], Madrid-Garcíaetal et al [51], Athavale et al [78], Birkun et al [85], Chen et al [90], Coskun et al [95], Gorelik et al [101], Henson et al [103], Hirosawa et al [104], Hirosawa et al [105], King et al [112], King et al [113], Kiyohara et al [114], Krusche et al [115], Kumari et al [117], Kusunose et al [119], Lahat et al [120], Samaan et al [141], Uz et al [149], Yeo et al [154], Harskamp et al [158] |
| Neurological Surgery |  |  | 5 | Alietal et al [22], Alietal et al [23], Guerraetal et al [35], Hopkinsetal et al [40], Mishra et al [127] |
| Obstetrics and Gynecology |  |  | 6 | Cohenetal et al [28], Guigueetal et al [36], Allahqoli et al [77], Lukac et al [123], Sütcüoğlu et al [146], Chervenak et al [156] |
| Ophthalmology |  |  | 15 | Antakietal et al [24], Caietal et al [26], Mihalacheetal et al [54], Moshirfaretal et al [55], Teebagyetal et al [71], Ali et al [76], Bernstein et al [84], Biswas et al [86], Delsoz et al [97], Delsoz et al [98], Hu et al [107], Lim et al [121], Lyons et al [124], Momenaei et al [128], Rojas-Carabali et al [138] |
| Orthopaedic Surgery |  |  | 9 | Cuthbertetal et al [29], Kungetal et al [46], Lumetal et al [50], Masseyetal et al [52], Saadetal et al [63], Duey et al [99], Kaarre et al [110], Kuroiwa et al [118], Mika et al [126] |
| Otolaryngology  – Head and Neck Surgery |  |  | 7 | Hochetal et al [38], Longetal et al [49], Ayoub et al [80], Bellinger et al [82], Chiesa-Estomba et al [91], Qu et al [131], Vaira et al [150] |
| Pathology |  |  | 4 | Hurleyetal et al [43], Benirschke et al [83], Cadamuro et al [87], Stevenson et al [145] |
| Pediatrics |  |  | 1 | Kao et al [111] |
| Plastic Surgery |  |  | 4 | Guptaetal et al [37], Humaretal et al [42], Hung et al [108], Copeland-Halperin et al [157] |
| Psychiatry and Neurology |  |  | 6 | Chenetal et al [27], Giannosetal et al [33], Schubertetal et al [64], Haemmerli et al [102], Hristidis et al [106], Wang et al [152] |
| Radiology |  |  | 16 | Bhayanaetal et al [25], Holmesetal et al [39], Patiletal et al [60], Barash et al [81], Fink et al [100], Kuckelman et al [116], Lyu et al [125], Nakaura et al [129], Rahsepar et al [132], Rao et al [133], Rau et al [135], Rogasch et al [137], Russe et al [139], Suthar et al [147], Ueda et al [148], Wagner et al [151] |
| Surgery |  |  | 4 | Ohetal et al [57], Abi-Rafeh et al [75], Samaan et al [142], Beaulieu-Jones et al [159] |
| Thoracic Surgery |  |  | 3 | Genceretal et al [32], Lietal et al [48], Shao et al [144] |
| Urology |  |  | 8 | Deebeletal et al [30], Caglar et al [88], Cakir et al [89], Cocci et al [93], Coskun et al [94], Davis et al [96], Whiles et al [153], Huynh et al [161] |
| General Practice |  |  | 19 | Friederichsetal et al [31], Gilsonetal et al [34], Meoetal et al [53], Oztermelietal et al [58], Roosetal et al [61], Rosołetal et al [62], Takagietal et al [69], Tanakaetal et al [70], Thirunavukarasuetal et al [72], Wangetal et al [74], Ayers et al [79], Clough et al [92], Johnson et al [109], Liu et al [122], Rao et al [134], Reese et al [136], Fang et al [160], Kung et al [162], Strong et al [163] |
| ETC  (clinical informatics, nursing) |  |  | 3 | Kanedaetal et al [44], Kumah-Crystaletal et al [45], Tairaetal et al [68] |

**Appendix Table 6. References of methods for evaluation based on test examinations**

| **Number of repeat measurements** | **N** | **Reference** |
| --- | --- | --- |
| 2 | 5 | Alietal et al [22], Schubertetal et al [64], Thirunavukarasuetal et al [72], Beaulieu-Jones et al [159], Strong et al [163] |
| 3 | 5 | Antakietal et al [24], Huangetal et al [41], Lietal et al [48], Longetal et al [49], Copeland-Halperin et al [157] |
| above 4 | 4 | Chenetal et al [27], Genceretal et al [32], Holmesetal et al [39], Hurleyetal et al [43] |
| **Prompt engineering** |  |  |
| Yes | 8 | Chenetal et al [27], Holmesetal et al [39], Huangetal et al [41], Lietal et al [48], Longetal et al [49], Schubertetal et al [64], Tanakaetal et al [70], Wangetal et al [74] |
| **Additional analysis** |  |  |
| Yes | 18 | Bhayanaetal et al [25], Caietal et al [26], Gilsonetal et al [34], Guptaetal et al [37], Holmesetal et al [39], Huangetal et al [41], Kungetal et al [46], Longetal et al [49], Madrid-Garcíaetal et al [51], Patiletal et al [60], Tanakaetal et al [70], Valdezetal et al [73], Wangetal et al [74], Copeland-Halperin et al [157], Beaulieu-Jones et al [159], Huynh et al [161], Kung et al [162], Fang et al [160] |
| **Difficulty** |  |  |
| Yes | 14 | Alietal et al [22], Alietal et al [23], Antakietal et al [24], Bhayanaetal et al [25], Caietal et al [26], Deebeletal et al [30], Gilsonetal et al [34], Lietal et al [48], Moshirfaretal et al [55], Rosołetal et al [62], Schubertetal et al [64], Suchmanetal et al [67], Takagietal et al [69] |

**Appendix Table 7. References of methods for evaluation by medical professionals**

| **Number of repeat measurements** | **N** | **Reference** |
| --- | --- | --- |
| 2 | 11 | Caglar et al [88], Cakir et al [89], King et al [112], King et al [113], Kuckelman et al [116], Lukac et al [123], Samaan et al [142], Shao et al [144], Yeo et al [154], Beaulieu-Jones et al [159], Strong et al [163] |
| 3 | 10 | Ali et al [76], Bellinger et al [82], Henson et al [103], Momenaei et al [128], Rao et al [133], Rao et al [134], Rogasch et al [137], Sütcüoğlu et al [146], Whiles et al [153], Copeland-Halperin et al [157] |
| above 5 | 5 | Birkun et al [85], Biswas et al [86], Kuroiwa et al [118], Rau et al [135], Russe et al [139] |
| **Number of evaluators** |  |  |
| 1 | 5 | Allahqoli et al [77], Hirosawa et al [105], Kiyohara et al [114], Kuroiwa et al [118], Kusunose et al [119] |
| 2 | 43 | Athavale et al [78], Ayoub et al [80], Barash et al [81], Bellinger et al [82], Birkun et al [85], Caglar et al [88], Cakir et al [89], Cocci et al [93], Coskun et al [94], Coskun et al [95], Duey et al [99], Gorelik et al [101], Hirosawa et al [104], Hung et al [108], Kao et al [111], King et al [112], King et al [113], Kuckelman et al [116], Lukac et al [123], Lyons et al [124], Lyu et al [125], Mika et al [126], Momenaei et al [128], Nakaura et al [129], Rahsepar et al [132], Rao et al [133], Rao et al [134], Rojas-Carabali et al [138], Russe et al [139], Samaan et al [141], Samaan et al [142], Sarbay et al [143], Sütcüoğlu et al [146], Ueda et al [148], Uz et al [149], Yeo et al [154], Angel et al [155], Chervenak et al [156], Copeland-Halperin et al [157], Harskamp et al [158], Fang et al [160], Beaulieu-Jones et al [159], Kung et al [162] |
| 3 | 13 | Ali et al [76], Ayers et al [79], Chen et al [90], Davis et al [96], Henson et al [103], Hristidis et al [106], Kumari et al [117], Lahat et al [120], Lim et al [121], O'Hagan et al [130], Reese et al [136], Rogasch et al [137], Suthar et al [147] |
| 4 | 3 | Fink et al [100], Kaarre et al [110], Mishra et al [127] |
| above 5 | 17 | Benirschke et al [83], Bernstein et al [84], Biswas et al [86], Cadamuro et al [87], Chiesa-Estomba et al [91], Clough et al [92], Haemmerli et al [102], Johnson et al [109], Krusche et al [115], Liu et al [122], Qu et al [131], Rau et al [135], Shao et al [144], Stevenson et al [145], Vaira et al [150], Whiles et al [153], Huynh et al [161] |
| **Prompt engineering** |  |  |
| Role-based prompting | 6 | Bernstein et al [84], Cadamuro et al [87], Hu et al [107], Kaarre et al [110], Nakaura et al [129], Angel et al [155] |
| Few shots learning | 2 | Bernstein et al [84], Kiyohara et al [114] |
| Explain context | 3 | Lim et al [121], Reese et al [136], Wang et al [152] |
| Template | 3 | Chen et al [90], Clough et al [92], Fink et al [100] |

**Appendix Table 8. Data Abstraction of Study Characteristics (Evaluation based on test examinations)**

| **No** | **Author** | **LLM model** | **Medical specialty** | **Test source** | **Question number** | **Performance** | **Language** | **Repeat measurement** | **Prompt engineering** | **Additional analysis** | **Difficulty** |
| --- | --- | --- | --- | --- | --- | --- | --- | --- | --- | --- | --- |
| 22 | Ali et al. | - GPT-3.5 - GPT-4 | Neurological Surgery | - Self-Assessment Neurosurgery Examinations (SANS) | 500 | - GPT-3.5: 73.4% - GPT-4: 83.4% | English | 2 | X | X | O |
| 23 | Ali et al. | - GPT-3.5 - GPT-4 - Bard | Neurological Surgery | - Self-Assessment Neurosurgery Exam (SANS) Indications Exam | 149 | - GPT-3.5: 62.4% - GPT-4: 82.6% - Bard: 44.2% | English | N/A | N/A | X | O |
| 24 | Antaki et al. | - GPT-3.5 - GPT-4 | Ophthalmology | - American Academy of Ophthalmology’s Basic and Clinical Science Course Self-Assessment Program(BCSC) - OphthoQuestions online question bank | 260 260 | - GPT-3.5:   - 55.8% (BCSC)  - 42.7% (OphthoQuestions)   - GPT-4:   - 59.4% (BCSC)  - 49.2% (OphthoQuestions) | English | 3 | X | X | O |
| 25 | Bhayana et al. | - GPT-3.5 | Radiology | - Canadian Royal College website - Multiple-choice bank for resident board examination preparation | 5 145 | - GPT-3.5: 69% | English | 1 | X | O | O |
| 26 | Cai et al. | - GPT-3.5 - GPT-4 - Bing Chat | Ophthalmology | - American Academy of Ophthalmology’s Basic and Clinical Science Course Self-Assessment Program(BCSC) | 250 | - GPT-3.5: 58.8% - GPT-4: 71.6% - Bing Chat: 71.2% | English | X | X | O | O |
| 27 | Chen et al. | - Bing Chat | Psychiatry and Neurology | - Glasgow Coma Scale (GSC) - Intracranial hemorrhage score (ICH) - Hunt & Hess (H&H) | 20 | - Average error rate:   - 10.0% (GCS)  - 13.0% (H&H)  - 27.5% (ICHe) | English | GCS: 5  H&H: 3  ICH: 4 | O | X | X |
| 28 | Cohen et al. | - GPT-3.5 | Obstetrics and Gynecology | - Hebrew OBGYN-‘Shlav-Alef’ examinations | 150 | - GPT-3.5: 38.7% | Hebrew English | N/A | X | X | X |
| 29 | Cuthbert et al. | - GPT-3.5 | Orthopaedic Surgery | - UK and Ireland In-Training Examination (UKITE) | 87 | - GPT-3.5: 35.8% | English | X | X | X | X |
| 30 | Deebel et al. | - GPT-3 | Urology | - American Urological Association Self-Assessment Study Program (AUA SASP) | 268 | - GPT-3:   - 42.3% (2021)  - 30.0% (2022) | English | N/A | X | X | O |
| 31 | Friederichs et al. | - GPT-3.5 | General Practice | - Berlin Progress Test | 400 | - GPT-3.5: 65.5% | Germany | N/A | X | X | X |
| 32 | Gencer et al. | - GPT-3.5 - GPT-4 | Thoracic Surgery | - Thoracic surgery theoretical exam | 15 | - GPT-3.5: 90.48% - GPT-4: 93.33% | Turkish | 7 | X | X | X |
| 33 | Giannos et al. | - GPT-3.5 Legacy - GPT-3.5 Default - GPT-4 | Psychiatry and Neurology | - Specialty Certificate Examination (SCE) | 69 | - GPT-3.5 Legacy: 42% - GPT-3.5 Default: 57% - GPT-4: 64% | N/A | X | X | X | X |
| 34 | Gilson et al. | - GPT-3 - Instruct GPT - GPT-3.5 | General Practice | - AMBOSS question bank - National Board of Medical Examiners (NBME) | 100 120 | - GPT-3.5:   - 64.4% (NBME step 1)  - 57.8% (NBME step 2)  - 44% (AMBOSS)   - Instruct GPT:   - 51.7% (NBME step 1)  - 52.9% (NBME step 2)  - 36% (AMBOSS)   - GPT-3:   - 25.3% (NBME step 1)  - 81.4% (NBME step 2)  - 80% (AMBOSS) | English | N/A | X | O | O |
| 35 | Guerra et al. | - GPT-4 | Neurological Surgery | - Congress of Neurological Surgeons Self-Assessment Neurosurgery Exam (SANS) | 591 | - GPT-4: 76.6% | English | N/A | N/A | X | X |
| 36 | Guigue et al. | - GPT-3.5 | Obstetrics and Gynecology | - Questions for the Parcours d'Accès Spécifique Santé | 885 | - GPT-3.5:   - 37.9% (OBGYN section)  - 33.0% (Whole test) | French | N/A | X | X | X |
| 37 | Gupta et al. | - GPT-3.5 | Plastic Surgery | - Plastic Surgery Inservice Training Examination (PSITE) | 242 | - GPT-3.5: 54.96% | English | N/A | X | O | X |
| 38 | Hoch et al. | - GPT-3.5 | Otolaryngology – Head and Neck Surgery | - Online learning platform to prepare for the German otolaryngology board certification | 2,576 | - GPT-3.5: 57% | Germany | N/A | X | X | X |
| 39 | Holmes et al. | - GPT-3.5 - GPT-4 - Bard - BLOOMZ | Radiology | - Examination on radiation oncology physics created by experienced medical physicist | 100 | - GPT-3.5: 53% - GPT-4: 75% - Bard: 33% - BLOOMZ: 43% | English | 5 | O | O | X |
| 40 | Hopkins et al. | - GPT-3.5 | Neurological Surgery | - Congress of Neurological Surgeons (CNS) Self-Assessment Neurosurgery (SANS) questions | 643 | - GPT-3.5: 53.2% | English | 1 | X | X | X |
| 41 | Huang et al. | - GPT-3.5 - GPT-4 | Family Medicine | - University of Toronto Department of Family and Community Medicine Progress Test | 108 | - GPT-3.5: 57.4% GPT-4: 82.4% | English | 3 | O | O | X |
| 42 | Humar et al. | - GPT-3.5 | Plastic Surgery | - The Plastic Surgery In-Service Examinations from 2018 to 2022 | 1129 | - GPT-3.5: 55.8% | English | N/A | N/A | X | X |
| 43 | Hurley et al. | - GPT-3.5 - GPT-4 - Bard | Pathology | - Internal Medicine BEST-TEST | 20 | - GPT-3.5: 40% - GPT-4: 87% - Bard: 55% | English | 10 | X | X | X |
| 44 | Kaneda et al. | - GPT-3.5 - GPT-4 | Nursing | - Japanese National Nursing Examination (JNNE) | 238 | - GPT-3.5: 59.9% - GPT-4: 79.7% | Japanese | N/A | N/A | X | X |
| 45 | Kumah-Crystal et al. | - GPT-3.5 | Clinical Informatics | - Questions from Mankowitz’s Clinical Informatics Board Review book | 254 | - GPT-3.5: 74% | English | N/A | N/A | X | X |
| 46 | Kung et al. | - GPT-3.5 - GPT-4 | Orthopaedic Surgery | - Orthopaedic In-Training Examination (OITE) | 360 | - GPT-3.5: 54.3%  GPT-4: 73.6% | English | N/A | X | O | X |
| 47 | Lewandowski et al. | - GPT-3.5 - GPT-4 | Dermatology | - Dermatology specialty certificate test | 358 | - GPT-3.5:   - 58.9% (Polish)  -66.5% (English)   - GPT4:   - 74.9%(Polish)  - 83.2%(English) | English Polish | N/A | N/A | X | X |
| 48 | Li et al. | - GPT-3.5 - GPT-4 - Claude - ChatGLM - FastChat | Thoracic Surgery | - Chinese National Senior Health Professional Technical Qualification Examination | 56 | - GPT-3.5:   - 38.00% (Zero-shot)  - 43.67% (Five-shot)   - GPT4:   - 48.00% (Zero-shot)  - 48.67% (Five-shot)   - Claude:   - 37.67% (Zero-shot)  - 41.67% (Five-shot)   - ChatGLM:   - 10.00% (Zero-shot)  - 6.33% (Five-shot)   - FastChat:   - 16.33% (Zero-shot)  - 12.00% (Five-shot) | Chinese | 3 | O (one shot, few shot learning) | X | O |
| 49 | Long et al. | - GPT-4 | Otolaryngology – Head and Neck Surgery | - Royal College of Physicians and Surgeons of Canada’s sample exam | 21 | - GPT-4: 92.06% | English | 3 | O | O | X |
| 50 | Lum et al. | - GPT-3.5 | Orthopaedic Surgery | - Orthopaedic In-Training Examination | 207 | - GPT-3.5: 47% | English | N/A | X | X | X |
| 51 | Madrid-García et al. | - GPT-3.5 - GPT-4 | Internal Medicine (Rheumatology) | - A dataset, RheumaMIR, extracted from the exams | 143 | - GPT-3.5: 66.43% - GPT-4: 93.71% | Spanish | N/A | N/A | O | X |
| 52 | Massey et al. | - GPT-3.5 - GPT-4 | Orthopaedic Surgery | - ResStudy orthopaedic examination question bank | 180 | - GPT-3.5: 29.4% - GPT-4: 47.2% | English | N/A | N/A | X | X |
| 53 | Meo et al. | - GPT-3.5 | General Practice | - A pool of multiple-choice questions (MCQs) from various medical textbooks | 100 | - GPT-3.5: 72% | English | N/A | X | X | X |
| 54 | Mihalache et al. | - GPT-3.5 | Ophthalmology | - Ophtho Questions practice question bank | 125 | - GPT-3.5: 46% | English | X | X | X | X |
| 55 | Moshirfar et al. | - GPT-3.5 - GPT-4 | Ophthalmology | - StatPearls question bank | 467 | - GPT-3.5: 55.5% - GPT-4 73.2% | English | N/A | X | X | O |
| 56 | Noda et al. | - GPT-3.5 - GPT-4 - Bard | Internal Medicine (Nephrology) | - Self-Assessment Questions for Nephrology Board Renewal | 99 | - GPT-3.5: 31.3% - GPT-4: 54.5% - Bard: 32.3% | Japanese | N/A | X | X | X |
| 57 | Oh et al. | - GPT-3.5 - GPT-4 | Surgery | - Korean general surgery board exams | 280 | - GPT-3.5: 46.8% - GPT-4: 76.4% | Korean | N/A | N/A | X | X |
| 58 | Oztermeli et al. | - GPT-3.5 | General Practice | - Medical Specialty Exam (MSE) | 1177 | - GPT-3.5: 54.3% ~ 70.9% | English | N/A | N/A | X | X |
| 59 | Passby et al. | - GPT-3.5 - GPT-4 | Dermatology | - Specialty Certificate Examination (SCE) in Dermatology | 84 | - GPT-3.5: 63% - GPT-4: 90% | English | N/A | N/A | X | X |
| 60 | Patil et al. | - GPT-4 - Bard | Radiology | - American College of Radiology’s Diagnostic Radiology In-Training (DXIT) examinations | 318 | - GPT-4: 87.11% - Bard: 70.44% | English | N/A | N/A | O | X |
| 61 | Roos et al. | - GPT-3.5 - GPT-4 - Bing Chat | General Practice | - German Medical State Examinations | 630 | - GPT4: 88.1% - Bing chat: 86.0% - GPT-3.5: 65.7% | German | N/A | X | X | X |
| 62 | Rosoł et al. | - GPT-3.5 - GPT-4 | General Practice | - Polish Medical Final Examination | 200 | - GPT-3.5:   - 56.6% (Polish)  - 58.3% (English)   - GPT4:   - 80.7% (Polish)  - 79.6% (English) | English Polish | N/A | X | X | O |
| 63 | Saad et al. | - GPT-4 | Orthopaedic Surgery | - Orthopaedic Fellow of the Royal College of Surgeons (FRCS Orth) Part A exam | 240 | - GPT-4: 67.5% | English | N/A | N/A | X | X |
| 64 | Schubert et al. | - GPT-3.5 - GPT-4 | Psychiatry and Neurology | - Question bank approved by the American Board of Psychiatry and Neurology | 1956 | - GPT-3.5: 66.8% - GPT4: 85% | English | 2(partially) | O | X | O |
| 65 | Shetty et al. | - GPT-3 - GPT-3.5 - GPT-4 | Dermatology | - MRCP UK Dermatology sample question dataset | 89 | - GPT-3: 48.31% - GPT3.5: 60.67% GPT4: 85.39% | English | N/A | X | X | X |
| 66 | Smith et al. | - GPT-3.5 legacy - GPT-4 - BARD - Bing Chat | Emergency Medicine | - ACEM primary examination | 240 | - GPT-3.5: 48.83% - GPT-4: 75.83% - Bard (PaLM): 63.75% - Bard (PaLM2): 65.83% - Bing Chat: 68.33% | English | N/A | N/A | X | X |
| 67 | Suchman et al. | - GPT-3 - GPT-4 | Internal Medicine (Gastroenterology) | - American College of Gastroenterology self-assessment tests | 455 | - GPT-3: 65.1% - GPT4: 62.4% | English | N/A | N/A | X | O |
| 68 | Taira et al. | - GPT-3.5 | Nursing | - Japanese National Nurse Examinations | 240 | - GPT-3.5:   - 75.1% (basic)  - 64.5% (general) | English  Japanese | N/A | X | X | X |
| 69 | Takagi et al. | - GPT-3.5 - GPT-4 | General Practice | - Japanese Medical Licensing Examination | 254 | - GPT-3.5: 50.8% - GPT-4: 79.9% | Japanese | N/A | X | X | O |
| 70 | Tanaka et al. | - GPT-3.5 - GPT-4 | General Practice | - Japanese National Medical Licensing Examination (JNMLE) | 290 | - GPT3.5:   - 52.8% (Japanese)  - 56.2% (English)  - 63.1% (few-shot learning)   - GPT-4:   - 78.6% (few-shot learning) | Japanese English (translate) | X | O | O | X |
| 71 | Teebagy et al. | - GPT-3.5 - GPT-4 | Ophthalmology | - Ophthalmology Knowledge Assessment Program (OKAP) | 180 | - GPT-3.5: 57% - GPT-4: 81% | English | N/A | X | X | X |
| 72 | Thirunavukarasu et al. | - GPT-3.5 | General Practice | - The Royal College of General Practitioners Applied Knowledge Test (AKT) | 674 | - GPT-3.5: 60.17% | English | 2 | X | X | X |
| 73 | Valdez et al. | - GPT-3 - GPT-4 | Internal Medicine (Rheumatology) | - 3rd party exam preparation website | 100 | - GPT-3: 62 % - GPT-4: 82% | English | N/A | X | O | X |
| 74 | Wang et al. | - GPT-3.5 - GPT-4 | General Practice | - China National Medical Licensing Examination (CNMLE) English National Medical Licensing Examination (ENMLE) | 100 | - GPT-3.5:   - 56% (CNMLE)  - 76% (ENMLE)   - GPT4:   - 84%(CNMLE)  - 86%(ENMLE) | English Chinese | N/A | O | O | X |

**Appendix Table 9. Data Abstraction of Study Characteristic (Evaluation by medical experts)**

| **No** | **Author** | **LLM model** | **Medical specialty** | **Purpose** | **Query Source** | **Number of queries** |  | **Evaluators** | **Performance** | **language** | **Repeat measurement** | **Role** | **Few shots learning** | **Explain Context** | **Template** |
| --- | --- | --- | --- | --- | --- | --- | --- | --- | --- | --- | --- | --- | --- | --- | --- |
| 75 | Abi-Rafeh et al. | - GPT-3.5 | Surgery | Diagnosis | - Hypothetical and standardized patient profiles | 16 | - Accuracy | - | - Accuracy:   - GPT-3.5: 85% | English | N/A | X | X | X | X |
| 76 | Ali et al. | - GPT-3.5 | Ophthalmology | Answering questions | - Questions developed by authors | 21 | - Accuracy | 3 | - GPT-3.5 Accuracy:   - 40% (correct)  - 25% (partailly correct)  - factually incorrect (25%) | English | 3 | X | X | X | X |
| 77 | Allahqoli et al. | - GPT-3.5 | Obstetrics and Gynecology | Diagnosis | - Cases from book named "100 Cases in Obstetrics and Gynecology" | 30 | - Accuracy | 1 | - Accuracy:   GPT-3.5: 90% | English | N/A | X | X | X | X |
| 78 | Athavale et al. | - GPT-3.5 - GPT-4 - Clinical Camel (LLaMA-based) | Internal Medicine (Cardiovascular Medicine) | Patient question answering | - Non-complex medical and administrative matters - Complex medical questions requiring subject matter expertise | 20 20 | - Accuracy | 2 | - Accuracy:   - GPT 3.5: 70% (Not complex), 45% (Complex)  - GPT-4: 100% (Not complex), 75% (Complex)  - Clinical Camel: 0% (Complex) | English | N/A | X | X | X | X |
| 79 | Ayers et al. | - GPT-3.5 | General Practice | Answering questions | - Questions from website r/AskDocs | 195 | - Preference - Quality of information - Empathy or bedside manner | 3 | - GPT-3.5:   - Preference: 78.6%  - Quality of information: 4.13/5  - Empathy: 3.65/5 | English | N/A | N/A | N/A | X | X |
| 80 | Ayoub et al. | - GPT-3.5 | Otolaryngology – Head and Neck Surgery | Patient education | - Questions developed by authors based on Clinical Practice Guidelines | 10 | - Patient Education Material Assessment Tool Score (24) - Relevance | 2 | - PEMAT-P score:   - GPT-3.5 68.2% | English | N/A | X | X | X | X |
| 81 | Barash et al. | - GPT-4 | Radiology | Clinical decision support | - Case data from hospital | 40 | - Clarity - Clinical relevance - Differential diagnosis | 2 | - GPT-4:   - Clarity: 4.8/5 (first reviewer), 4.6/5 (second reviewer)  - Clinical relevance: 4.5 (first reviewer), 4.4/5 (second reviewer)  - Differential diagnosis: 4.9/5 (first reviewer), 4.9/5 (second reviewer) | English | N/A | X | X | X | X |
| 82 | Bellinger et al. | - GPT-3.5 | Otolaryngology – Head and Neck Surgery | Patient education | - Questions developed by authors | 5 | - Accuracy - Currency - Readability - Quality - Understandability and actionability | 2 | - GPT-3.5:   - Accuracy: 4.19/5  - Currency: 4.31/5 | English | 3 | X | X | X | X |
| 83 | Benirschke et al. | - GPT-4 | Pathology | Answering questions | - Questions previously sent to pathology residents from health care providers | 61 | - Accuracy - Completeness - Potential medical mistake | 11 | - GPT-4:   - Accuracy: 98%  - Completeness: 82%  - Near-zero potential for medical harm: 97% | English | N/A | X | X | X | X |
| 84 | Bernstein et al. | - GPT-3.5 | Ophthalmology | Answering questions | - Questions from an online advice forum | 200 | - Presence of incorrect information - Alignment with perceived consensus in the medical community - Likelihood to cause harm - Extent of harm | 8 | - Presence of incorrect information:   - No (77.4%)   - Likelihood of possible harm:   - 0.9% (Definitely harmful)  -12.6% (Potentially harmful)   - Extent of possible harm:   - 2.9% (Severe harm)  - 14.8% (Mild/moderate harm) | English | N/A | O | O | X | X |
| 85 | Birkun et al. | - Bing Chat | Internal Medicine (Cardiovascular Medicine) | Answering questions | - Questions based on google trend | 1 | - Satisfaction - Readability | 2 | - Bing Chat Completely/partially satisfied:   - English: 53.2%  - Gambia: 37.3%  - India: 48.6% | English Gambia India | 20 | X | X | X | X |
| 86 | Biswas et al. | - GPT-3.5 | Ophthalmology | Answering questions | - Questions on webpage of the Association of British Dispensing Opticians and the College of Optometrists | 11 | - Quality of information | 5 | - GPT-3.5 Quality of information:   - 24% (very good)  - 48.7% (good)  - 21.8% (acceptable)  - 3.6% (poor)  - 1.8% (very poor) | English | 5 | X | X | X | X |
| 87 | Cadamuro et al. | - GPT-4 | Pathology | Answering questions | - Fictional clinical cases were defined by WG-AI members | 10 | - Relevance - Correctness - Helpfulness - Safety | 7 | - Not specific result reported | English | N/A | O | X | X | X |
| 88 | Caglar et al. | - GPT-3.5 | Urology | Answering questions | - Questions regarding pediatric urology on the websites | 137 | - Accuracy - Reproducibility | 2 | - GPT-3.5:   - Accuracy: 92%  - Reproducibility: 93.8% - 100% | English | 2 | X | X | X | X |
| 89 | Cakir et al. | - GPT-3.5 | Urology | Answering questions | - Questions about urolithiasis on the websites | 93 | - Accuracy - Reproducibility | 2 | - GPT-3.5:   - Accuracy: 94.6%  - Reproducibility: 90.0% - 100% | English | 2 | X | X | X | X |
| 90 | Chen et al. | - GPT-3.5 | Internal Medicine (Oncology) | Answering questions | - Concordance with National Comprehensive Cancer Network guidelines | 104 | - Concordance | 3 | - Concordance: - - GPT-3.5: 98% | English | N/A | X | X | X | O |
| 91 | Chiesa-Estomba et al. | - GPT-3.5 | Otolaryngology – Head and Neck Surgery | Clinical decision support | - Clinical scenarios developed by authors | 6 | - Agreement with experts | 10 | - Agreement:   - GPT-3.5: 3.4/5 | English | N/A | X | X | X | X |
| 92 | Clough et al. | - GPT-3.5 | General Practice | Generating discharge summaries | - Case vignettes developed by authors | 25 | - Acceptability | 5 | - Acceptability:   - GPT-3.5: 100% | English | N/A | X | X | X | O |
| 93 | Cocci et al. | - GPT-3.5 | Urology | Clinical decision support | - Case data from hospital | 100 | - Accuracy - Comprehensiveness - Clarity - Quality - Readability | 2 | - Appropriate   - GPT-3.5: 52% | English | N/A | X | X | X | X |
| 94 | Coskun et al. | - GPT-3.5 | Urology | Patient information | - Website of the European Association of Urology | 59 | - Accuracy (F1 scores) - Similarity (Cosine similarity) - Quality (General Quality Score) | 2 | - GPT-3.5   - Accuracy: 0.426  - Similarity: 0.609  - Quality: 3.62 | English | N/A | N/A | N/A | X | X |
| 95 | Coskun et al. | - GPT-3.5 - GPT-4 - BARD - Bing Chat | Internal Medicine (Rheumatology) | Patient information | - Questions established by previous research | 23 | - Accuracy - Completeness | 2 | - Accuracy:   - GPT-3.5: 100%  - GPT-4: 100%  - BARD: 73.91%  - Bing chat: 73.91% | English | N/A | X | X | X | X |
| 96 | Davis et al. | - GPT-3.5 | Urology | Answering questions | - Questions about urological symptoms and treatments by Google Trends | 18 | - Appropriateness - Readability - Output Quality | 3 | - Appropriateness:   - GTP-3.5: 77.8% | English | N/A | X | X | X | X |
| 97 | Delsoz et al. | - GPT-3.5 - GPT-4 | Ophthalmology | Diagnosis | - Cases data from online database | 20 | - Accuracy | - | - Accuracy:   - GPT-3.5: 60%  - GPT-4: 85% | English | N/A | X | X | X | X |
| 98 | Delsoz et al. | - GPT-3.5 | Ophthalmology | Diagnosis | - Cases data from online database | 11 | - Accuracy | - | - Accuracy   - GPT-3.5: 72.7% | English | N/A | X | X | X | X |
| 99 | Duey et al. | - GPT-3.5 - GPT-4 | Orthopaedic Surgery | Answering questions | - Questions from NASS guidelines for antithrombotic therapies | 12 | - Accuracy - Over-conclusiveness - Supplemental information - Incompleteness | 2 | - Accuracy   - GPT-3.5: 33%  - GPT-4: 92%   - Over-conclusiveness   - GPT-3.5: 50%  - GPT-4: 8%   - Supplemental information   - GPT-3.5: 67%  - GPT-4: 92%   - Incompleteness   - GPT-3.5: 33%  - GPT-4: 33% | English | N/A | X | X | X | X |
| 100 | Fink et al. | - GPT-3.5 - GPT-4 | Radiology | Oncologic reasoning capabilities | - Free-text radiology reports from hospital | 424 | - Accuracy - Factual correctness | 4 | - Accuracy:   - GPT-3.5: 84.0%  - GPT-4: 98.6% | English | N/A | X | X | X | O |
| 101 | Gorelik et al. | - GPT-4 | Internal Medicine (Gastroenterology) | Clinical decision support | - Clinical scenarios | 20 | - Accuracy - Compliance with guidelines | 2 | - GPT-4:   - Accuracy: 85% - Compliance: 90% | English | N/A | X | X | X | X |
| 102 | Haemmerli et al. | - GPT-3.5 | Psychiatry and Neurology | Clinical decision support | - Case data from hospital | 10 | - Agreement with experts | 7 | - GPT-3.5:   - Diagnosis quality: Poor (median 3)  - Treatment recommendation quality: Good (medical 7)  - Therapy regimen quality: Good (median 7) | English | N/A | X | X | X | X |
| 103 | Henson et al. | - GPT-3.5 | Internal Medicine (Gastroenterology) | Answering questions | - Questions developed by authors | 23 | - Appropriateness - Specificity | 3 | - Appropriateness:   - 29.0% (Completely appropriate)  - 62.3% (Mostly appropriate)  - 8.7% (Mostly inappropriate)   - Specificity:   - 78.3% (Some specific guidance)  - 21.7% (Only generic information) | English | 3 | X | X | X | X |
| 104 | Hirosawa et al. | - GPT-3.5 | General Internal Medicine | Differential diagnoses | - Vignettes developed by the author | 30 | - Accuracy | 2 | - Accuracy:   - GPT-3.5: 93.3% | English | N/A | X | X | X | X |
| 105 | Hirosawa et al. | - Bard | General Internal Medicine | Differential diagnosis | - Case reports and mock cases | 82 | - Accuracy | 1 | - Accuracy:   - 40.2% (As top diagnosis)  - 53.7% (Within top 5)  - 56.1% (Within top 10) | English | N/A | X | X | X | X |
| 106 | Hristidis et al. | - GPT-3.5 | Psychiatry and Neurology | Answering questions | - Questions developed by authors based on Alzheimer’s Disease Knowledge Scale | 60 | - Relevance - Currency of information - Reliability - Objectivity | 3 | - GPT-3.5:   - Relevance: 96.7%  - Currency of information: 0%  - Reliability of information: No source (96.7%)  - Objectivity: 100% | English | N/A | X | X | X | X |
| 107 | Hu et al. | - GPT-4 | Ophthalmology | Diagnosis | - Cases data from online | 10 | - Appropriateness - Accuracy | - | - GPT-4:   - Appropriateness: 83.3% (Overall)  - Appropriateness simulated: 70% (Patient), 100% (Family physician (100%), 80% (Junior ophthalmologist) | English | N/A | O | X | X | X |
| 108 | Hung et al. | - GPT-3.5 | Plastic Surgery | Patient education | - Questions developed by authors | 12 | - Accuracy | 2 | - Accuracy:   - GPT-3.5: 50% | English | N/A | X | X | X | X |
| 109 | Johnson et al. | - GPT-3.5 | General Practice | Answering questions | - Questions developed by physicians | 284 | - Accuracy - Completeness | 33 | - GPT-3.5:   - Accuracy: 4.8/6 - Completeness: 2.5/3 | English | N/A | X | X | X | X |
| 110 | Kaarre et al. | - GPT-4 | Orthopaedic Surgery | Answering questions | - Questions from previous research | 20 | - Accuracy - Completeness - Adaptiveness | 4 | - GPT-4   - Correctness: 1.69/2 (Patient), 1.66/2(Medical doctors)  - Completeness: 1.51/2 (Patients), 1.64/2 (Medical doctors)  - Adaptiveness: Patients (1.75/2), Medical doctors (1.73/2) | English | N/A | O | X | X | X |
| 111 | Kao et al. | - GPT-3.5 | Pediatrics | Clinical decision support | - Questions developed by authors | 8 | - Overall grade | 2 | - GPT-3.5's overall grade: C | English | N/A | X | X | X | X |
| 112 | King et al. | - GPT-3.5 - GPT-4 | General Internal Medicine | Patient education | - Questions from esteemed medical societies and institutions | 98 | - Accuracy - Reproducibility | 2 | - GPT-3.5 Accuracy:   - General: 85.7  - Cardiology: 83.3%  - Gastroenterology: 53.3%   - GPT-4 Accuracy:   - General: 94.6%  - Cardiology: 83.3%  - Gastroenterology: 60%   - - Neurology: 66.7% | English | 2 | X | X | X | X |
| 113 | King et al. | - GPT-3.5 - GPT-4 | Internal Medicine (Cardiovascular Medicine) | Answering questions | - Questions from medical societies, renowned medical institutions | 107 | - Accuracy - Reproducibility | 2 | - Accuracy   - GPT-3.5: 78.5%  - GPT-4: 83.2%   - Reproducibility - GPT-3.5: 98.1% - GPT-4: 100% | English | 2 | X | X | X | X |
| 114 | Kiyohara et al. | - GPT-3.5 - GPT-4 - Bard | Internal Medicine (Cardiovascular Medicine) | distinguish between specific diseases | - Cases from journals and abstracts in Japan | 66 | - Accuracy | 1 | - Accuracy:   - GPT-3.5: 52% (Zero-shot), 52%(Few-shot)  - GPT-4: 58% (Zero-shot), 61%(Few-shot)   - - Bard: 47% (Zero-shot) | Japanese | N/A | X | O | X | X |
| 115 | Krusche et al. | - GPT-4 | Internal Medicine (Rheumatology) | Diagnosis | - Data set from previous research | 132 | - Accuracy | 33 | - Accuracy   - GPT-4: 35% (top diagnosis), 60% (top 3 diagnoses) | English | N/A | X | X | X | X |
| 116 | Kuckelman et al. | - Bing Chat | Radiology | Patient information | - Questions developed by authors and Chatbot | 30 | - Accuracy - Completeness | 2 | - Bing Chat   - Accuracy: 93% - Completeness: 65% | English | 2 | X | X | X | X |
| 117 | Kumari et al | - GPT-3.5 - Bard - Bing Chat | Internal Medicine (Hematology) | Solving hematology-related cases | - Cases developed by authors | 50 | - Accuracy | 3 | - Accuracy:   - GPT-3.5: 3.15/5 - Bard: 2.23/5 - Bing Chat: 1.98/5 | English | N/A | X | X | X | X |
| 118 | Kuroiwa et al. | - GPT-3.5 | Orthopaedic Surgery | Self-diagnosis | - Questions developed by authors | 25 | - Accuracy Reproducibility | 1 | - GPT-3.5 accuracy:   - Carpal tunnel syndrome: 100%  - Cervical myelopathy: 4%  - Lumbar spinal stenosis: 96%  - Knee osteoarthritis: 64%  - Hip osteoarthritis: 68% | English | 5 days per 5 people | X | X | X | X |
| 119 | Kusunose et al. | - GPT-3.5 | Internal Medicine (Cardiovascular Medicine) | Answering questions | - | 31 | - Accuracy | 1 | - GPT-3.5Accuracy:   - Japanese: 64.5%  - English: 58.0% | Japanese English | N/A | X | X | X | X |
| 120 | Lahat et al. | - GPT-3.5 | Internal Medicine (Gastroenterology) | Answering questions | - Questions gathered from websites | 110 | - Accuracy - Clarity - Efficacy | 3 | - GPT-3.5 Accuracy:   - Treatment: 3.9/5  - Symptom: 3.4/5  - Diagnostic test: 3.7/5   - GPT-3.5 Clarity:   - Treatment: 3.9/5  - Symptom: 3.7/5  - Diagnostic test: 3.7/5   - GPT-3.5 Efficacy   - Treatment: 3.3/5  - Symptom: 3.2/5  - Diagnostic test: 3.5/5 | English | N/A | X | X | X | X |
| 121 | Lim et al. | - GPT-3.5 - GPT-4 - Bard | Ophthalmology | Answering questions | - Questions gathered from websites | 31 | - Accuracy - Comprehensiveness | 3 | - Accuracy:   - GPT-4: 80.6%  - GPT-3.5: 61.3%  - Google Bard: 54.8% | English | N/A | X | X | O | X |
| 122 | Liu et al. | - GPT-3.5 | General Practice | Clinical decision support | - Best Practice Advisories from hospital | 7 | - Usefulness - Acceptance - Relevance - Understanding - Workflow - Bias - Inversion - Redundancy | 5 | - GPT-3.5:   - Usefulness: 2.7//5  - Acceptance: 1.8/5 - Relevance: 3.6/5 - Understanding: 3.9/5 - Workflow: 1.5/5 - Bias: 1.4/5 - Inversion: 1.6/5 - Redundancy: 1.7/5 | English | N/A | X | X | X | X |
| 123 | Lukac et al. | - GPT-3.5 | Obstetrics and Gynecology | Supporting therapy planning | - Case data from hospital | 10 | - Answering score system | 2 | - Agreement:   - GPT-3.5: 64.2% | Germany | 2 | X | X | X | X |
| 124 | Lyons et al. | - GPT-4 - Bing Chat | Ophthalmology | differential diagnosis | - Vignettes developed by authors | 44 | - Accuracy of diagnosis - Accuracy of timing range | 2 | - Accuracy of diagnosis   - Bing Chat: 77% (Within top 3)  - GPT-4: 93% (Within top 3)   - Accuracy of timing range   - Bing Chat: 84% - GPT-4: 98% | English | N/A | X | X | X | X |
| 125 | Lyu et al. | - GPT-3.5 - GPT-4 | Radiology | Report for Patients | - Screening reports collected from the Atrium Health Wake Forest Baptist clinical database | 62 | - Overall score - Accuracy - Completeness | 2 | - GPT-3.5: 55.2% (Original prompt), 77.2% (optimized prompt) - GPT-4: 73.6% (original prompt), 96.8% (optimized prompt) | English | N/A | X | X | X | X |
| 126 | Mika et al. | - GPT-3.5 | Orthopaedic Surgery | Answering questions | - Questions gathered from websites | 10 | - Accuracy | 2 | - GPT-3.5 accuracy:   - 20% (Excellent)  - 40% (Satisfactory requiring minimal clarification)  - 30% (Satisfactory requiring moderate clarification)  - 10% (Unsatisfactory requiring substantial clarification) | English | N/A | X | X | X | X |
| 127 | Mishra et al. | - GPT-3.5 | Neurological Surgery | Answering questions | - Common conditions for which neurosurgical interventions are used | 40 | - Quality of contents | 4 | - Quality:   - GPT-3.5: 44.2/75 | English | N/A | X | X | X | X |
| 128 | Momenaei et al. | - GPT-4 | Ophthalmology | Answering questions | - Questions developed by authors | 88 | - Appropriateness - Readability | 2 | - Appropriateness:   - 84.6% (RD)  - 92% (MH)  - 91.7% (ERM) | English | 3 | X | X | X | X |
| 129 | Nakaura et al. | - GPT-2 - GPT-3.5 - Bing Chat | Radiology | Generating radiology reports | - Image findings from the “Radiology Review Manual, 8th ed” | 28 | - Overall quality | 2 | - Accuracy:   - GPT-2: 0.21(Top1), 0.46(Top5)  - GPT-3.5: 0.54(Top1), 0.89(Top5)  - Bing Chat accuracy: 0.54(Top1), 0.96(Top5) | English | N/A | O | X | X | X |
| 130 | O'Hagan et al. | - GPT-3.5 - GPT-4 | Dermatology | Answering questions | - Questions developed by authors | 25 | - Accuracy | 3 | - Accuracy:   - GPT-3.5: 4.29/5 - GPT-4: 4.53/5 | English | N/A | X | X | X | X |
| 131 | Qu et al. | - GPT-4 | Otolaryngology – Head and Neck Surgery | Differential diagnosis and management | - Vignettes developed by authors | 20 | - Agreement with experts | 11 | - Agreement (range of median score)   - 3.00 - 5.00 / 5.00 (differential diagnosis)  - 3.00 - 5.00 / 5.00 (treatment plans) | English | N/A | X | X | X | X |
| 132 | Rahsepar et al. | - GPT-3.5 - Bard | Radiology | Answering questions | - Nonexpert questions regarding lung cancer prevention, screening, and terminology | 40 | - Accuracy | 2 | - Accuracy:   - GPT-3.5: 70.8% - Bard: 51.7% | English | N/A | X | X | X | X |
| 133 | Rao et al. | - GPT-3.5 - GPT-4 | Radiology | Clinical decision support | - The ACR Appropriateness Criteria | 7 | - Accordance with ACR guidelines | 2 | - Screening:   - GPT-3.5: 88.9%(SATA), 1.83/2(OE)  - GPT-4: 98.4%(SATA), 1.83/2(OE)   - Pain:   - GPT-3.5: 58.3%(SATA), 1.125/2(OE)  - GPT-4: 77.7%(SATA), 1.666/2(OE) | English | 3 | X | X | X | X |
| 134 | Rao et al. | - GPT-3.5 | General Practice | Clinical decision support | - Clinical vignettes selected from the Merck Sharpe & Dohme Clinical Manual | 36 | - Concordant with MSD Manual answer | 2 | - Accuracy:   GPT-3.5: 71.7% | English | 3 | X | X | X | X |
| 135 | Rau et al. | - GPT-3.5 - GPT-4 - accGPT | Radiology | Clinical decision support | - Clinical cases created based on the ACR appropriateness criteria | 50 | - Appropriateness | 6 | - Appropriateness:   - GPT-3.5: 70%  - GPT-4: 79%   - - accGPT: 83% | English | 6 | X | X | X | X |
| 136 | Reese et al. | - GPT-4 | General Practice | differential diagnosis | - Case reports from NEJM | 75 | - Accuracy | 3 | - Accuracy: - - GPT-4: 38.7% | English | N/A | X | X | O | X |
| 137 | Rogasch et al. | - GPT-3.5 | Radiology | Answering questions | - Questions developed by authors | 13 | - Appropriateness - Helpfulness - Empathetic - Inconsistent between trials - Validity of references | 3 | - GPT-3.5:   - Appropriate: 92% - Useful: 96% | Germany | 3 | X | X | X | X |
| 138 | Rojas-Carabali et al. | - GPT-3.5 - GPT-4 | Ophthalmology | Diagnosis | - Cases based on Standardization of Uveitis Nomenclature (SUN) guidelines | 25 | - Accuracy | 2 | - Accuracy:   - GPT-3.5: 60% (Top diagnosis), 64% (Top 3 diagnosis)  - GPT-4: 60% (Top diagnosis), 72% (Top 3 diagnosis) | English | N/A | X | X | X | X |
| 139 | Russe et al. | - GPT-3.5 - GPT-4 - FraCChat 3.5 - FraCChat 4 | Radiology | Clinical decision support | - Knowledge base from the AOOTA Fracture and Dislocation Classification Compendium | 100 | - Accuracy | 2 | - Accuracy:   - GPT-3.5: 3% - GPT-4: 2% - FraCChat 3.5: 48% - FraCChat 4: 71% | English | 5 | X | X | X | X |
| 140 | Zúñiga Salazar et al. | - GPT-3.5 - Bard - Bing Chat | Emergency Medicine | Determine medical emergencies | - Patients’ questions posted to Reddit’s community r/AskDocs | 176 | - Concordance | - | - Accuracy:   - GPT-3.5: 77%  - Bard: 87%   - - Bing Chat: 82% | English | N/A | X | X | X | X |
| 141 | Samaan et al. | - GPT-3.5 | Internal Medicine (Gastroenterology) | Answering questions | - Questions from professional societies and institutions | 91 | - Accuracy | 2 | - Accuracy:   - 24.2% (Comprehensive)  - 48.4% (Correct but inadequate)  - 14.3% (Mixed)  - 13.2% (Completely incorrect) | Arabic  English | N/A | X | X | X | X |
| 142 | Samaan et al. | - GPT-3.5 | Surgery | Answering questions | - Questions from the American Society for Metabolic and Bariatric Surgery and MedlinePlus Medical Encyclopediaibility | 151 | - Accuracy - Reproducibility | 2 | - GPT-3.5:   - Accuracy: 86.8% - Reproducibility: 90.7% | English | 2 | X | X | X | X |
| 143 | Sarbay et al. | - GPT-3.5 | Emergency Medicine | triage prediction | - Case developed by authors and from ESI handbook | 50 | - Concordance | 2 | - GPT-3.5:   - Sensitivity: 57.1% - Specificity: 34.5%  - PPV: 38.7%  - NPV: 52.6% - F1 score: 0.461 | English | N/A | X | X | X | X |
| 144 | Shao et al. | - GPT-3.5 | Thoracic Surgery | Patient education | - Questions developed by authors | 37 | - Appropriateness | 35 | - Appropriateness:   - GPT-3.5: 92% (both English and Chinese) | English Chinese | 2 | X | X | X | X |
| 145 | Stevenson et al. | - GPT-3.5 - Bard | Pathology | Clinical decision support | - Cases developed by authors | 15 | - Accuracy - Safety | 5 | - Accuracy:   - GPT-3.5: 33.3% - Bard: 20.0% | English | N/A | X | X | X | X |
| 146 | Sütcüoğlu et al. | - GPT-3.5 | Obstetrics and Gynecology | Answering questions | - Questions developed by authors based on European Society of Human Reproduction and Embryology's guidelines | 25 | - Appropriateness | 2 | - Appropriate:   - GPT-3.5: 76% | English | 3 | X | X | X | X |
| 147 | Suthar et al. | - GPT-4 | Radiology | Differential Diagnosis | - diagnostic quizzes presented in the AJNR's "Case of the Month." | 140 | - Accuracy | 3 | - Accuracy:   - GPT-4: 57.86% | English | N/A | X | X | X | X |
| 148 | Ueda et al. | - GPT-4 | Radiology | Diagnosis | - Educational Diagnosis Please Quizzes | 313 | - Concordance - Legitimacy | 2 | - Accuracy:   - GPT-4: 54% | English | N/A | X | X | X | X |
| 149 | Uz et al. | - GPT-3.5 | Internal Medicine (Rheumatology) | Obtaining Information | - Keywords based on google trend | 4 | - Reliability - Usefulness | 2 | - Reliability:   - 5.62/7 (Osteoarthritis)   - Usefulness:   - 5.87/7(ankylosing spondylitis) | English | N/A | X | X | X | X |
| 150 | Vaira et al. | - GPT-4 | Otolaryngology – Head and Neck Surgery | Answering questions | - Questions developed by authors | Question : 144 Clinical Scenarios : 15 | - Accuracy - Completeness | 18 | - GPT-4:   - Accuracy: 87.2% - Completeness: 73% | English | N/A | X | X | X | X |
| 151 | Wagner et al. | - GPT-3 | Radiology | Answering questions | - Questions developed by authors | 88 | - Accuracy | - | - Accuracy:   -GPT-3: 67% | English | N/A | X | X | X | X |
| 152 | Wang et al. | - GPT-4 | Psychiatry and Neurology | Primary Screening | - Dat from institute (Dementia Bank) | 174 | - | - | - GPT-4:   - Sensitivity: 0.8636  - specificity: 0.9487   - - AUC: 0.9062 | English | N/A | X | X | O | X |
| 153 | Whiles et al. | - GPT-3.5 | Urology | Patient question answering | - Questions developed by authors based on general urologic principles | 13 | - Appropriateness - Quality | 5 | - GPT-3.5:   - Appropriateness: 60%  - Quality: 16.8 | English | 3 | X | X | X | X |
| 154 | Yeo et al. | - GPT-3.5 | Internal Medicine (Gastroenterology) | Answering questions | - Questions form professional societies and institutions | 164 | - Accuracy - Reproducibility | 2 | - Accuracy:   - GPT-3.5: 47.2%(Cirrhosis), 50%(HCC) | English | 2 | X | X | X | X |

**Appendix Table 10. Data Abstraction of Study Characteristic (Both)**

| **No** | **Author** | **LLM model** | **Medical specialty** | **Purpose** | **Query Source** | **Number of queries** |  | **Evaluators** | **Performance** | **language** | **Repeat measurement** | **Role** | **Few shots learning** | **Explain Context** | **Template** |
| --- | --- | --- | --- | --- | --- | --- | --- | --- | --- | --- | --- | --- | --- | --- | --- |
| 75 | Abi-Rafeh et al. | - GPT-3.5 | Surgery | Diagnosis | - Hypothetical and standardized patient profiles | 16 | - Accuracy | - | - Accuracy:   - GPT-3.5: 85% | English | N/A | X | X | X | X |
| 76 | Ali et al. | - GPT-3.5 | Ophthalmology | Answering questions | - Questions developed by authors | 21 | - Accuracy | 3 | - GPT-3.5 Accuracy:   - 40% (correct)  - 25% (partailly correct)  - factually incorrect (25%) | English | 3 | X | X | X | X |
| 77 | Allahqoli et al. | - GPT-3.5 | Obstetrics and Gynecology | Diagnosis | - Cases from book named "100 Cases in Obstetrics and Gynecology" | 30 | - Accuracy | 1 | - Accuracy:   GPT-3.5: 90% | English | N/A | X | X | X | X |
| 78 | Athavale et al. | - GPT-3.5 - GPT-4 - Clinical Camel (LLaMA-based) | Internal Medicine (Cardiovascular Medicine) | Patient question answering | - Non-complex medical and administrative matters - Complex medical questions requiring subject matter expertise | 20 20 | - Accuracy | 2 | - Accuracy:   - GPT 3.5: 70% (Not complex), 45% (Complex)  - GPT-4: 100% (Not complex), 75% (Complex)  - Clinical Camel: 0% (Complex) | English | N/A | X | X | X | X |
| 79 | Ayers et al. | - GPT-3.5 | General Practice | Answering questions | - Questions from website r/AskDocs | 195 | - Preference - Quality of information - Empathy or bedside manner | 3 | - GPT-3.5:   - Preference: 78.6%  - Quality of information: 4.13/5  - Empathy: 3.65/5 | English | N/A | N/A | N/A | X | X |
